# CNTO 530 Increases Expression of HbA and HbF in Murine Models of β-Thalassemia and Sickle Cell Anemia

**DOI:** 10.2174/138920113805219449

**Published:** 2013-02

**Authors:** Dorie A Makropoulos, Ram Achuthanandam, Justin Avery, Krista Wilson, Kerry Brosnan, Andrew Miller, Thomas Nesspor, Denise Chroscinski, Mindi Walker, Devon Egenolf, ChiChi Huang, Peter J Bugelski

**Affiliations:** 1 Biologics Toxicology, Center of Excellence in Biotechnology, Centocor R&D, Radnor PA, USA; 2 Ace Animals, Inc., Boyertown, PA, USA

**Keywords:** Erythropoiesis, Flow Cytometry, Fusion protein, Hematology, Ion Exchange Chromatography, Pharmacodynamics.

## Abstract

CNTO 530 is an erythropoietin receptor agonist MIMETIBODY^TM^ construct. CNTO 530 has been shown to be active in a number of rodent models of acquired anemia (e.g. renal insufficiency and chemotherapy induced anemia). We investigated the efficacy of CNTO 530 in murine models of β-thalassemia and sickle cell anemia (Berkeley mice). β- thalassemic mice are deficient in expression of α-globin chain and heterozygous mice are characterized by a clinical syndrome similar to the human β-thalassemia intermedia. Berkeley mice are knocked out for murine alpha and beta globin and are transgenic for human alpha, beta (sickle) and gamma globin genes. Berkeley mice thus express human sickle hemoglobin A (HbS) and can also express human fetal hemoglobin. These mice express a severe compensated hypochromic microcytic anemia and display the sickle cell phenotype. To test the effectiveness of CNTO 530, mice from both genotypes received a single subcutaneous (s.c.) dose of CNTO 530 or darbepoetin-α (as a comparator) at 10,000 U/kg, a dose shown to cause a similar increase in reticulocytes and hemoglobin in normal mice. Hematologic parameters were evaluated over time. CNTO 530, but not darbepoetin-α, increased reticulocytes, red blood cells and total hemoglobin in β- thalassemic mice. In Berkeley mice CNTO 530 showed an increase in reticulocytes, red blood cells, F-cells, total hemoglobin and fetal hemoglobin. In conclusion, CNTO 530 is effective in murine models of β-thalassemia and sickle cell anemia. These data suggest that CNTO 530 may have beneficial effects in patients with genetically mediated hemoglobinopathies.

## INTRODUCTION

### Background

Erythropoiesis, the formation of red blood cells (RBC) from multipotent stem cells in the bone marrow, is an exquisitely regulated process in which the glycoprotein hormone erythropoietin (EPO) plays a central role [1]. Recently, a novel long-acting erythropoietin receptor agonist (EPO-R) has been described [2]. CNTO 530 is a 57 kD glycoprotein homodimer MIMETIBODY^TM^ construct with two copies of EMP-1 (an erythropoietic peptide [3]) presented near the N-terminus. CNTO 530, like EPO, binds EPO-R and stimulates erythropoiesis. However, CNTO 530 bears no amino acid sequence homology to EPO. 

β-thalassemia and sickle cell anemia (SCA) are two of the most common hemoglobinopathies [4, 5]. The adult hemoglobin molecule (HbA) is composed of two α-type and two β-type globin chains. In β-thalassemia, underproduction of to the β-globin chain results in precipitation of the unpaired α-chains, which in turn, triggers the destruction of RBC and red cell precursors in the bone marrow [6]. Numerous β-globin mutations can result in β-thalassemia. Depending on the mutation, the degree of underproduction of β-globin chain varies. In some patients, the imbalance of globin chain production is minimal and they remain symptom free (thalassemia minor). In others, there is a greater imbalance resulting in transfusion dependent anemia (thalassemia intermedia and thalassemia major). In these latter types, increased destruction of RBC and repeated transfusions leads to hemochromatosis. 

In contrast, SCA is the result of a unique point mutation in the β globin gene that results in production of sickle hemoglobin (HbS) [5]. In sickle cell disease, individuals homozygous for the mutant β-globin gene produce no HbA and exclusively HbS. However, they retain some production of fetal hemoglobin (HbF). The solubility of the deoxygenated form of HbS is less than HbA. This lower solubility results in precipitation of HbS in the red cells, causing deformity of the cells (sickle cells) and leading to destruction of RBC, formation of micro-thrombi and sever pain. Exacerbation of this process is referred to as sickle cell crisis and can lead to infarctions in numerous tissues, especially the spleen, liver and brain [7].

### Rationale

In baboons, rhEPO has been shown to increase the percent of reticulocytes containing HbF from 1-2% to as high as 50% [8] and rhEPO has been shown to have some, albeit limited, benefit in β-thalassemia and SCA (see below). This beneficial effect is believed, at least in part, to be derived from an up-regulation of gamma-globin chain utilization, leading to production of increased levels of fetal hemoglobin (HbF). The purpose of this study was to determine if CNTO 530 has beneficial effects in murine models of β-thalassemia and SCA.

## MATERIALS AND METHODS

### Test Articles 

CNTO 530 and formulation buffer was supplied by Centocor, R&D (Radnor PA, Lot # TN101806). Darbepoetin-α (Aranesep^TM^, Amgen, Thousand Oaks, CA) was purchased commercially. Phosphate buffered saline was purchased from Invitrogen (Carlsbad, CA). 

Because CNTO 530 and darbepoetin-α have different molecular weights and different affinity for EPO-R, doses were expressed as UT-7 Units/kg. UT-7 units were determined from an *in vitro* assay described previously [2]. Briefly, using epoetin-α (mw = 34 kD) with an activity of 120 IU/μg as a standard, the EC_50_ for each test article for mediating proliferation of UT-7_EPO_ cells and their respective molecular weight were used to calculate UT-7 units using the following equation: 

UT-7 Units/µg = (Mol wt of ERA)*C/(EC_50_ for ERA),

where C = (120 Units/µg)*(EC_50_ for rHuEPO)/34 kD

This gave values of 199 U/µg for darbepoetin-α and 30 U/µg for CNTO 530. 

### Mice 

Female C57BL/6 mice were obtained from Ace Animals, Boyertown, PA. Male and female Th3+/C57BL/6 (heterozygous) (β-thalassemic mice) [9] were obtained from a colony maintained in the pathogen-free vivarium at Centocor R&D, Inc., Radnor PA. Founder Th3+/C57BL/6 mice for the colony was obtained from M Weiss, University of Pennsylvania. The breeding stock was maintained as heterozygotes for a deletion of both the b1 and b2 globin genes. Th3+/C57BL/6 were selected for study based on a pale visual appearance and gross splenomegally in comparison with their Th3-/C57BL/6 littermates. 

Hb^atm1Paz^ Hbb^tm1Tow^ Tg(HBA-HBBs)41Paz/J (Berkeley) mice [10] were bred at Ace Animals (Boyertown, PA). Founder mice for the transgenic colony were obtained from Jackson Laboratories (Stock number 003342, Bar Harbor, ME) under a nonexclusive bailment agreement with the Regents of the University of California through the EOL Berkeley National Laboratory. As recommended by Jackson Laboratories, dams heterozygous for murine β-globin (non-sickle) were bred to murine β-globin knockout (sickle) males. All mice were genotyped to ensure that they were murine β-globin knockout and transgene + (Tg/Tg or Tg/o). Only mice that were murine β-globin homozygous knockout and expressed the transgene were used in these experiments. 

All mice were group housed in filter-topped plastic shoebox style cages. The animals were individually identified with ear tags, placed at least 1 week prior to the start of the study. All mice were maintained in the pathogen-free vivarium at Centocor R&D, Inc., Radnor PA. The Institutional Animal Care and Use Committee at Centocor approved all associated procedures. 

### Pharmacodynamics

Mice received a single subcutaneous (s.c.) dose of CNTO 530 or darbepoetin-α at 10,000 U/kg. Control mice received an equivalent volume of the saline vehicle or formulation buffer. Blood was collected from mice anesthetized with a CO_2_ mixture via open chest cardiac puncture into commercially prepared EDTA coated microtubes. Hematology analyses were performed on whole blood using an ADVIA® 120 hematology analyzer (Siemens Medical Solutions Diagnostics, Tarrytown NY, USA).

Ion exchange chromatorgraphic analysis of HbF was performed after the method of Morin and Barton [11]. Briefly, 50 μL fresh whole blood was lysed in 200 μL distilled H_2_O containing 0.1% Triton-X 100 and 200 mM KCN and 1 mL adsorption buffer was added. (The adsorption buffer contained 200 mM Bis-Tris acetate (pH 4.5), 200 mM KCN and a trace amount of trichlorobutanol as a preservative.) One cm disposable mini-columns were packed with 1 mL Sephadex CM-50 in adsorption buffer. The column was allowed to drain under minimal vacuum, the packing covered with a glass frit and washed with adsorption buffer. One ml of sample was layered on the packing and the column washed with 2 aliquots of adsorption buffer. The column was eluted with 2 mL aliquots of elution buffer and fractions were collected under gravity. (The elution buffer contained 100 mM Bis-Tris acetate (pH 6), 4.8 g/L magnesium acetate, 200 mM KCN, and a trace amount of trichlorobutanol as a preservative.) Aliquots of the collected fractions were transferred to a 96 well plate and the OD 415 (Soret peak for hemoglobin (Hgb)) read on a Molecular Devices SpectraMax 340PC (Sunnyvale, CA). Pre- and post-dose HbF ratio was calculated using the value for the peak fraction. To calculate HbF concentration, 250 μL of the remaining whole blood lysate was diluted to 2 mL and serial 2 fold dilutions prepared in adsorption buffer. Aliquots of these dilutions (0.33 mL) were transferred to a 96 well plate and the OD 415 read. These values were plotted as a function of dilution of total Hgb (measured by the automated hematology analyzer) and linear regression analysis was used to calculate an extinction coefficient for each mouse. This value was used with the OD of the HbF peak to calculate the concentration of HbF. 

Flow cytometric analysis of RBC and reticulocytes expressing HbF was performed after the method of B Davis and K Davis (Current Protocols in Cytometry, 2004). Briefly, ~25x10^6^ RBC were fixed with 1 mL cold 0.05% glutaraldehyde in phosphate buffered saline (PBS) for 10 minutes. The cells were washed with 2 mL 0.1% bovine serum albumin (BSA) 0.1% sodium azide in PBS and permeabilized in 500 μL 0.1% Triton-X 100 for 3-5 minutes, washed and resuspended in 500 μL BSA-PBS. Ten μL aliquots were stained with anti-HbF antibodies (5 μL in 80 μL BSA-PBS) in a 96 well round bottom plate for 15 minutes (Murine monoclonal anti-human HbF, clone HbF-1 (12) Cy5 (TRI-COLOR^®^, TC), Invitrogen). The cells were washed and resuspended in 200 μL thiazole orange (Retic-Count™ Reticulocyte Reagent System, Becton Dickinson Biosciences, San Jose, CA) for 15-30 minutes. Staining was controlled with a Fetal Hemoglobin Control Kit (Fetaltrol), Invitrogen, and BD Retic-Count™ Control Kit (Tri-Level Control). Data were acquired on a Becton Dickinson FACSCalibur. Monodisperse cells were gated on the basis of forward and side scatter. Cells stained with HbF-1 were counted as HbF+ and cells stained with thiazole orange were counted as reticulocytes. Data are expressed as %Total RBC HbF+ and %Reticulocytes HbF+. 

### Data Analysis and Statistics

Data are expressed as mean ± standard deviation. Statistical significance was determined by *t*-test or analysis of variance with Bonferroni’s correction for multiple comparisons. When the data were not normally distributed or if there was unequal variance, significance was determined by Mann-Whitney Rank Sum or analysis of variance on ranks with Dunn’s correction for multiple comparisons. P values <0.05 were accepted as statistically significant. 

## RESULTS 

### Characterization of β-thalassemic Mice

The results of the comparison of Th3+/C57BL/6 (β-thalassemic) with C57BL/6 (normal) are shown in (Table **1** and Table **2**). Hematologic analysis confirmed dysregulated erythropoiesis in these mice with dramatically decreased RBC, Hgb, and hematocrit (Hct), by up to 40% compared to normal C57Bl/6 mice. Reticulocyte counts were elevated approximately 6 fold concurrent with decreased mean cell volume (MCV), mean cell hemoglobin (MCH), and mean cell hemoglobin concentration (MCHC). Moreover, red cell distribution width (RDW), a measure of size heterogeneity, was increased by approximately 3 fold. In addition, splenomegaly was observed consistent with increased hematopoiesis. Thus, Th3+/C57BL/6 mice can be described as exhibiting a marked microcytic, hypochromic, regenerative anemia and ineffective erythropoiesis. 

### Characterization of Berkeley Mice

The results of the comparison of Berkeley (sickle cell) with C57BL/6 (normal) mice are shown in (Table **1** and Table **2**). Compared to normal C57Bl/6 mice, Berkeley mice showed a number of changes in RBC parameters such as a decrease in RBC, Hgb, Hct, MCH and MCHC with an increase in retic and RDW. Although the mean MCV were not different, there was a difference in variance, probably reflecting fragmentation of RBC. These data indicate that the Berkeley mice show a poorly compensated, normocytic, hypochromic anemia.

### Pharmacodynamics of CNTO 530 and Darbepoetin-α 

**Fig. (1):**
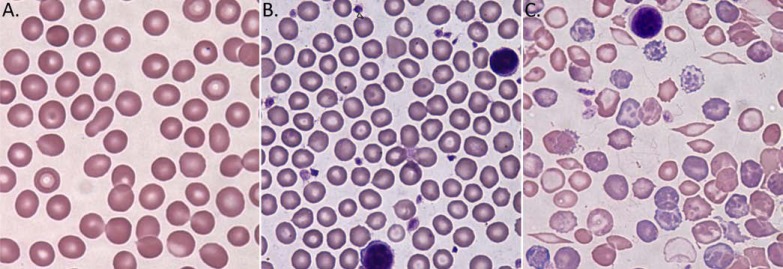
Peripheral blood smeer at 100x. (**A**) C57BL/6 mouse (**B**) TH3+/C57BL/6 mouse (**C**) Berkeley mouse. Wright Giemsa staining of Peripheral blood smear at 100x. The image acquisition system consisted of a Nikon Eclipse 80i microscope (Nikon Inc., Melville, NY) Nikon 100x oil immersion objective, Evolution TM MP color camera (Media Cybernetics, Inc., Bethesda, MD) and Image-Pro Plus V 5.1 software (Media Cybernetics).

To determine the effects of CNTO 530 or darbepoetin-α in normal, β-thalassemic and sickle cell mice, all were given a single s.c. dose of 10,000 U/kg of CNTO 530 or darbepoetin-α. As shown in (Fig. **1a**), this dose of CNTO 530 and darbepoetin-α caused a similar peak reticulocytosis in normal C57BL/6 mice. However, the duration of the elevation of reticulocytes was longer with CNTO 530. The longer duration of reticulocytosis was reflected in a greater and longer-lived increase in RBC (Fig. **1b**) and Hgb (Fig. **1c**). 

**Fig. (2):**
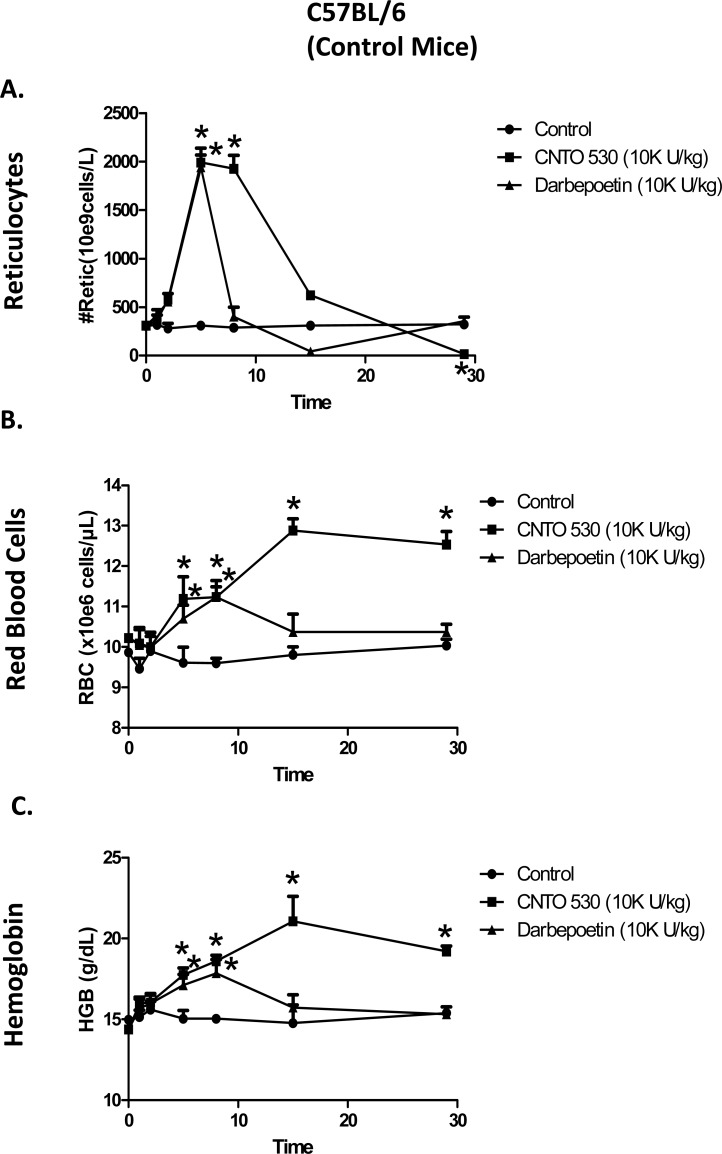
Comparative effects of CNTO 530 and Darbepoetin-α on (**A**) Reticulocytes, (**B**) RBC, and (**C**) Hgb in normal C57BL/6 (control) mice. Asterisk (*) indicates statistically greater than control, ANOVA with Bonferroni or ANOVA on Ranks, Dunn’s correction, (P<0.05).

In β-thalassemic mice, compared to darbepoetin-α, CNTO 530 caused a greater and longer-lived increase in reticulocytes in Th3+/C57BL/6 mice (Fig. **2a**). Again, as seen in normal mice, the longer duration of reticulocytosis was reflected in a greater and longer-lived increase in RBC (Fig. **2b**) and Hgb (Fig. **2c**). 

**Fig. (3):**
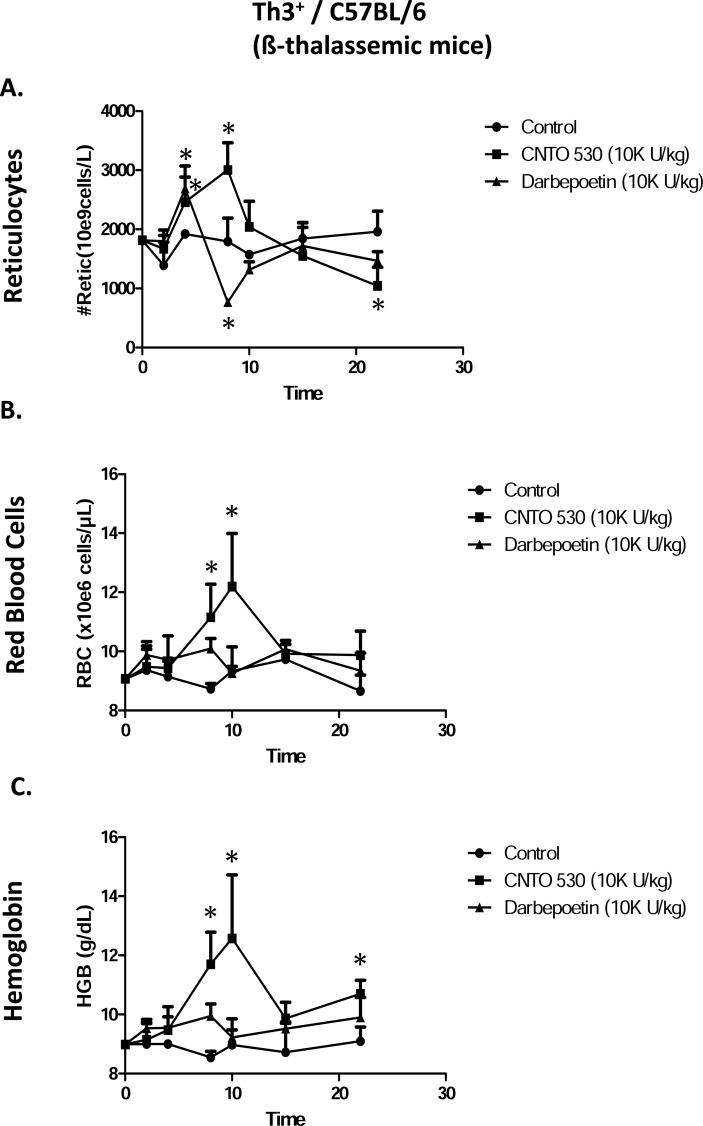
Comparative effects of CNTO 530 and Darbepoetin-α on (**A**) Reticulocytes, (**B**) RBC, and (**C**) Hgb in ß-thalassemic mice. Asterisk (*) indicates statistically greater than control, ANOVA with Bonferroni or ANOVA on Ranks, Dunn’s correction, (P<0.05).

**Fig. (4):**
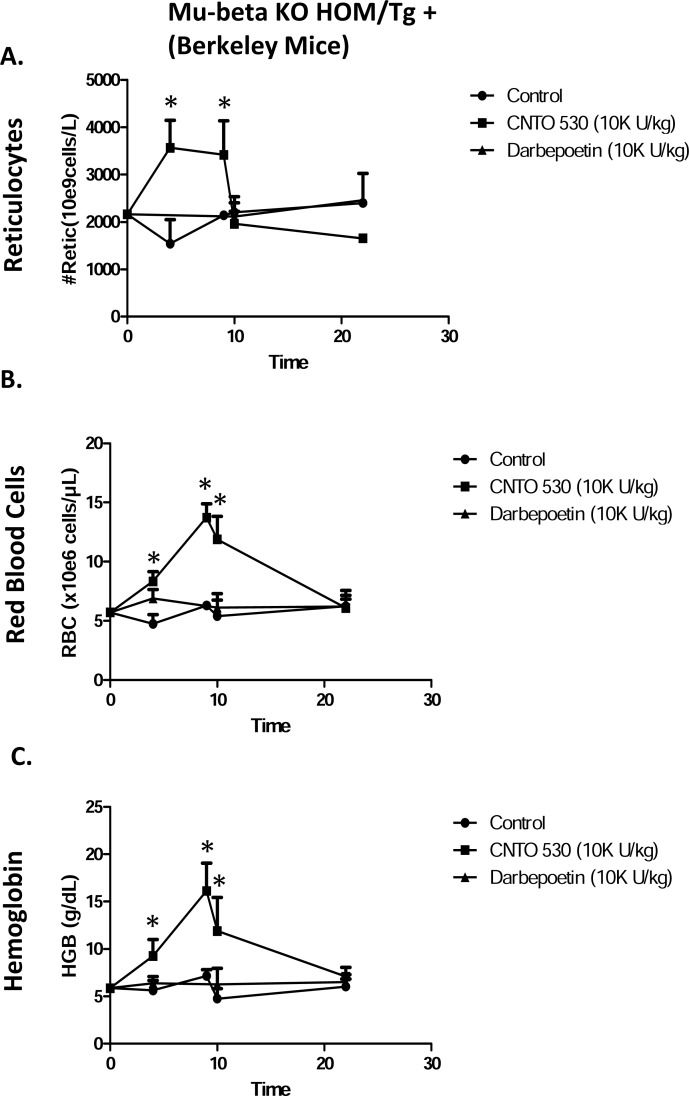
Comparative effects of CNTO 530 and Darbepoetin- α on (**A**) Reticulocytes, (**B**) RBC, and (**C**) Hgb in Berkeley mice. Asterisk (*) indicates statistically greater than control, ANOVA with Bonferroni or ANOVA on Ranks, Dunn’s correction, (P<0.05).

In sickle cell mice, CNTO 530 caused an increase in reticulocytes (Fig. **3a**). In contrast, darbepoetin-α had no effect on reticulocytes in these mice. The increase in reticulocytes in the CNTO 530 treated mice was reflected in an increase RBC (Fig. **3b**) and Hgb (Fig. **3c**). Again, in contrast, darbepoetin-α had no effect on these parameters. To determine the effects of CNTO 530 and darbepoetin-α on expression of HbF, blood samples collected 10 days after dosing with 10,000 U/kg were analyzed by ion exchange chromatography. There was a statistically significant increase total Hgb (Fig. **4a**) and in expression of HbF (Fig. **4b**) in the CNTO 530 treated but not the darbepoetin-α treated mice. These blood samples were also analyzed by flow cytometry for enumeration of HbF+ reticulocytes and RBC. The gating strategy for measuring F+ reticulocytes and F+ RBC in sickle cell mice is shown in Fig. **4c** (control), Fig. **4d** (CNTO 530 treated), and Fig. **4e** (darbepoetin-α). The effects of CNTO 530 on %F+ reticulocytes and %F+ RBC are shown in (Fig. **4f** and **4g**). CNTO 530, but not darbepoetin-α, caused a statistically significant increase in % F reticulocytes and %F+ RBC.

## DISCUSSION

In this report, murine models of ß-thalassemia and SCA have been characterized and the comparative effects of a novel erythropoietin receptor agonist fusion protein and darbepoetin-α described. Although both test articles were active in normal mice at the doses tested, their effects were attenuated in the models and only CNTO 530 increased RBC and Hgb. CNTO 530 also increased expression of HbF in the SCA mice. Although the increase in HbF was small, in SCA, patients with HbF levels lower than 8-9% are more anemic than those with HbF levels higher than 8-9% [12] suggesting that relatively small increases in expression of HbF may be beneficial. 

Prior to embarking on the pharmacodynamic experiments, the anemia in Th3+/C57Bl/6 and Berkeley mice was characterized. Originally described by Yang *et al.* [9], Th3+/C57BL/6 mice are heterozygous for a deletion of both the beta-1 and beta-2 globin gene. As shown in (Table **1**), these mice showed a decrease in RBC, Hgb, Hct, MCV and MCH and an increase in HDW. Examination of peripheral blood smears showed Howell-Jolly bodies, target cells, and moderate anisocytosis. Additionally, the mice were splenomegalic. WBC indices were modestly elevated while platelet indices were unremarkable. These findings are similar to those described in human ß-thalassemia intermedia/major [4]. Berkeley mice were originally described by Pászty *et al.* [10]. These mice are knocked out for murine alpha and beta globin and are transgenic for human alpha, beta(sickle) and gamma globin genes. Thus, they express human hemoglobin A (HbAsickle) (or sickle hemoglobin, HbS) and can also express human HbF. As shown in (Table **1**), these mice showed a marked reticulocytosis, decreased RBC, Hgb and Hct and decreased MCH. Examination of peripheral blood smears showed numerous target cells, fragmented red cells, and sickle cells. In addition, the mice were splenomegalic and WBC and platelet indices were markedly affected. These findings are similar to those described in human SCA [5]. Thus, Th3+/C57Bl/6 and Berkeley mice were considered good models to test the effects of CNTO 530. 

In embryonic and fetal life, alternate forms of Hgb, HbE and HbF are expressed. These forms of Hgb are composed of α-type globin chains and the embryonic (epsilon-globin) or fetal (gamma-globin) chains [13]. Although these forms of Hgb predominate in embryonic and fetal life, respectively, they are sequentially down regulated and HbE is normally not expressed and HbF is expressed at low levels postnatally. In Berkeley mice, the human fetal ^G^γ- and ^A^γ-globin genes were inserted as a 39kb Kpn1 fragment (with the human adult δ and β^S^ globin genes) in their normal genomic context [10]. As reported by Pászty *et al.* [10], newborn Berkeley mice expressed appreciable amounts of human HbF and trace amounts of HbE but as adults HbF and HbE were not detected. In contrast, in our study trace amounts of HbF were detectable in control adult Berkeley mice using ion exchange chromatography. Increased expression of HbF is considered important as these forms of Hgb can alleviate anemia in β-thalassemia and SCA and may decrease sickle crisis [14, 15]. Erythropoietin receptor agonists either alone, or more commonly in combination with hydroxy urea have been shown to have beneficial activity in ß-thalassemia and SCA, [16-21]. This is probably, at least in part, through increased expression of HbF [22-25], but others have failed to find an effect of rhEPO on HbF expression [26].

The mechanism by which CNTO 530 increases HbF in Berkeley mice is unknown. As reviewed by Mahjan *et al.* [27], although control of gamma globin gene expression has been the subject of intensive study the normal control mechanisms remain incompletely understood. Moreover, as reviewed by Mabaera *et al.* [14], neither the molecular mechanisms nor target molecules for any of the over 70 pharmacologic agents that can increase expression of HbF have been verified. Regarding EPO-R agonists, in addition to its well known growth factor, anti-apoptotic and differentiation effects on early erythroid precursors [28, 29], EPO has also been shown to express a number of other effects including cytoprotective [30] and improved iron and glucose metabolism [31, 32].

Although CNTO 530 utilizes the same receptor and signal transduction systems as epoetin-α [2], the EMP-1 pharmacophore of CNTO 530 engages EPO-R in a manner distinct from epoetin-α[33]. Thus, CNTO 530 may have novel effects beyond its function as a long-lived EPO-R agonist. This hypothesis is supported by the work of Sathyanarayana *et al.* [34]_ENREF_34 who showed that CNTO 530 increased expression of podocalyxin X by erythroblasts and caused a sustained expansion of a Kit^neg^CD71^high^Ter119^neg^ erythroid progenitor pool in murine bone marrow. Thus, the effects of CNTO 530 in β-thalassemic and SCA mice may depend on a unique engagement of the EPO-R resulting in pharmacologic effects beyond stimulation of erythropoiesis.

## CONCLUSIONS

In conclusion, this single dose pharmacodynamic study has shown that CNTO 530 is active in murine models of β-thalassemia and SCA. CNTO 530 showed superior activity compared to darbepoetin-a and therefore may have other beneficial effects in addition to being a long-lived EPO-R agonist. Thus, testing CNTO 530 in β-thalassemia and SCA may be warranted.

## Figures and Tables

**Fig. (5):**
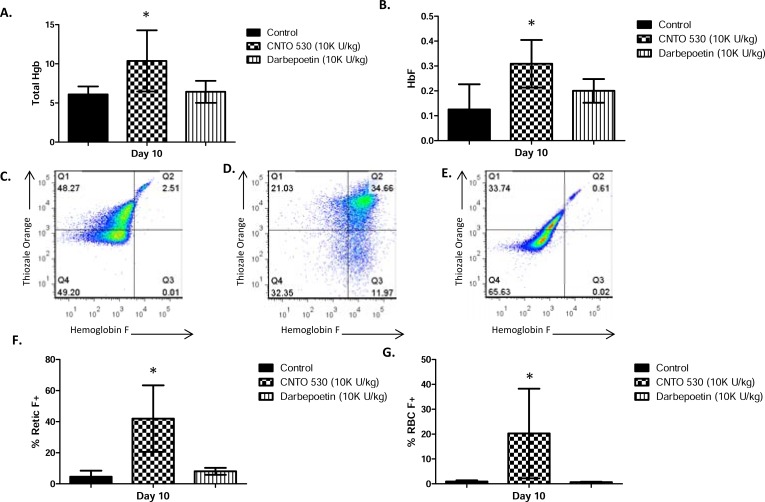
Ion Exchange Chromatographic Analysis of comparative effects of CNTO 530 and darbepoetin-α on Hgb from Berkeley mice on Day 10. (**A**) Total Hgb and (**B**) HbF. Flow cytometric analysis of HbF+ retics and RBC in Berkeley mice on Day 10. Representative results are shown for control and each treatment. Retics were gated based on staining with thizole orange (Quadrants 1 and 2). HbF+ retics (Quadrant 2) and RBC (Quadrant 3) were gated based on staining for HbF. Numbers in corners are mean fluorescence intensity (MFI). (**C**) PBS Control (**D**) CNTO 530 (**E**) Darpepoetin-α. Comparative effects of CNTO 530 on (**F**) retics stained for HbF and (G) RBC stained for HbF.

**Table 1.** Differences in the Erythron Between C57Bl/6, β-Thalassemic, and Berkeley Mice. Asterisk (*) Indicates Statistically Different from Control, *t*-test, (P<0.05).

Mouse Strain	Statistics	Reticulocytes				Mature Erythrocytes							
		Retic (x10^9^cells/L)	CHr (pg)	Retic_ RDW (%)	Retic_ HDW (g/dL)	RBC (x10^6^/uL)	HGB (g/dL)	HCT (%)	RDW (%)	MCV (fL)	MCH (pg)	MCHC (g/dL)	HDW (g/dL)
**C57BL/6 n=30**	**Mean Std. Dev.**	304.59 ± 47.98	16.11 ± 0.30	11.61 ± 0.66	1.91 ± 0.09	9.74 ± 0.31	15.22 ± 0.43	53.42 ± 1.74	12.39 ± 1.18	54.86 ± 1.04	15.63 ± 0.29	28.49 ± 0.40	1.36 ± 0.04
**TH3+/C57BL/6 n=34**	**Mean Std. Dev.**	1751.45* ± 388.28	13.97* ± 0.55	17.39* ± 1.11	3.64* ± 0.20	9.09* ± 0.65	8.94* ± 0.58	36.64* ± 1.95	34.51* ± 2.79	40.13* ± 1.72	9.81* ± 0.61	24.45* ± 1.18	4.27* ± 0.34
**Berkeley n=18**	**Mean Std. Dev.**	1880.09* ± 453.38	13.93* ± 1.38	17.23* ± 2.34	3.35* ± 0.49	5.27* ± 1.12	5.59* ± 1.03	27.01* ± 3.87	25.16* ± 3.52	52.42 ± 7.87	10.91* ± 2.18	20.68* ± 1.57	3.57* ± 0.27

**Table 2.** Differences in Leukocytes and Platelets Between C57Bl/6, β-Thalassemic, and Berkeley Mice. Asterisk (*) Indicates Statistically Different from Control, *t*-test, (P<0.05).

Mouse Strain	Statistics	Leukocytes				Platelets			
		WBC (x10^3^/µL)	Neuts (x10^3^cells/µL)	Lymphs (x10^3^cells/µL)	Mono (x10^3^cells/µL)	Plt (x10^3^/µL)	MPV (fL)	PDW (%)	MPC (g/dL)
**C57BL/6 n=30**	**Mean Std. Dev.**	10.64 ± 3.08	0.98 ± 0.35	9.10 ± 2.65	0.12 ± 0.06	1195.27 ± 227.57	5.73 ± 0.46	48.72 ± 2.16	23.13 ± 1.68
**TH3+/C57BL/ 6n=34**	**Mean Std. Dev.**	17.52* ± 5.23	1.19 ± 0.50	15.63* ± 4.90	0.25 ± 0.13	1370.21 ± 188.77	6.54 ± 0.78	59.33* ± 14.09	23.22 ± 2.09
**Berkeley n=18**	**Mean Std. Dev.**	25.48* ± 19.06	1.92* ± 0.92	22.42* ± 17.86	0.13 ± 0.15	898.56 ± 384.24	9.10* ± 1.19	83.02* ± 14.97	19.27* ± 1.61
